# New Opportunities to Strengthen Medicaid Managed Care Network Adequacy Standards

**DOI:** 10.1001/jamahealthforum.2023.3194

**Published:** 2023-10-06

**Authors:** Jane M. Zhu, Kranti C. Rumalla, Daniel Polsky

**Affiliations:** Division of General Internal Medicine, Oregon Health & Science University, Portland; Feinberg School of Medicine, Northwestern University, Chicago, Illinois; Department of Health Policy and Management, Bloomberg School of Public Health, Johns Hopkins University, Baltimore, Maryland

In April 2023, the US Centers for Medicare & Medicaid Services (CMS) proposed new standards for Medicaid managed care relating to network adequacy.^1^ The proposed rule requires that states establish appointment wait time standards within federal guidelines and enforce these standards by using independent secret shopper surveys. These recommendations represent a notable shift from prior network adequacy approaches,^2^ which have had little measurable effect. As state and federal regulators increasingly turn toward network adequacy standards as an important tool to facilitate access to care, these proposed rules introduce new opportunities for considering how health plan networks can meet enrollee needs.

Under capitated payment models, managed care plans are steeped in trade-offs between access and cost. The choice between narrower vs broader networks is 1 example, with health plans exerting influence over costs—at the potential risk of reducing access—by contracting with specific sets of clinicians and facilities to deliver services to their enrollees. A potential benefit of narrower networks is to help insurers direct patients to lower-cost and higher-quality clinicians, and research^3^ suggests that patients often tolerate this trade-off in exchange for lower premiums. In Medicaid, where enrollees do not pay premiums, these trade-offs may exhibit differently (eg, managed care contracts may be less likely to include areas where costs are higher). Research^4^ suggests that narrow networks are associated with less access to specialists, particularly those delivering behavioral health or cancer care. When a managed care plan’s health care network is so narrow as to be inadequate, enrollees may not receive the benefits promised.

Network adequacy standards may help regulators curb these undesirable consequences and safeguard access to care, but their efficacy has been limited. First, they have been imprecisely defined and inconsistently operationalized. Traditionally, network adequacy standards have been defined broadly. Since 2020, Medicaid managed care plans have been allowed to adopt any quantitative standard, such as maximum travel distance or time, minimum provider ratios, and/or maximum wait times, with no federal quantitative standard and no dedicated enforcement mechanisms. As a result, network adequacy standards across Medicaid managed care plans are highly variable.^5^ Considering the massive growth in Medicaid managed care in recent years,^1^ this opacity has led to a patchwork of rules, with health plans regulating and monitoring their own networks with minimal accountability. Enforcement of network adequacy standards is exceedingly difficult, leading to wide gaps in perceived access to care. In a 2019 California secret shopper study,^6^ Medicaid patients were able to schedule urgent care appointments with only 54% and routine appointments with 64% of listed primary care clinicians.

Second, measures of health care networks rely heavily on information that is out of date, inaccurate, or incomplete. Across insurance markets, including Medicaid, audits of health plan directories suggest error rates as high as 50% on phone numbers, addresses, and other contact information. Studies^7,8^ have also found the prevalence of “phantom” clinicians (ie, those listed in plan directories but who do not actually see patients according to claims billing) to be on the order of 25% to 60%, with substantial variation across plans and specialties.

Third, network adequacy standards applied at the health plan level are fundamentally unable to account for the complexity of health care professionals’ contracting arrangements across health plans and markets. The typical primary care physician contracts with a median of 8 health plans,^9^ with specialists often participating in far more networks. Thus, network adequacy standards applied at the health plan level cannot take into account clinicians’ capacity and availability within the same market, much less across markets. Clinicians serving enrollees in numerous insurer products may be unable to adequately meet the needs of all networks with which they contract.

Fourth, network adequacy standards are applied unevenly, which presents a challenge to meaningful regulation and oversight. Network adequacy regulations differ across insurance markets, but even within the same market segments, standards vary widely across states in their operational aspects and definitions. For example, California’s Medicaid managed care program mandates specialist and mental health standards, while Florida requires only provider-to-enrollee ratios.^5^ While variation in network adequacy standards is to be expected based on local contexts, such as supply of health care professionals, rurality, geography, and market conditions, considerable differences even in how network adequacy is conceptualized suggests that there currently lacks a unified understanding of what constitutes sufficient access, and how to measure it.

Taken together, these challenges point to a critical problem: current network adequacy standards do not appear to correlate with patient access, experience, or outcomes,^2,10^ which in turn has hampered regulatory action and enforcement. In this setting, the new Medicaid managed care proposed rules^1^ call for maximum appointment wait time standards for both primary care and mental health professionals, defining a maximum number of days a patient should have to wait to be seen. For example, a plan would need to ensure a primary care appointment within 30 days, an urgent care appointment within 2 days, and routine behavioral health care within 14 days. These measures have the advantage. compared with maximum travel distance or time and minimum provider ratios, of directly measuring access as experienced by enrollees. For accountability, CMS proposes audits of managed care plan compliance through annual secret shopper surveys by an independent third party. This information, along with results from annual consumer experience surveys, will be published online for public transparency.

Several additional considerations may help optimize the impact of network adequacy regulations and expand beyond Medicaid managed care. This includes additional transparency and accountability using consumer surveys to monitor patient experience and dimensions of availability, accessibility, and equity, including language and cultural competencies. Surveys can also obtain information on aspects of health care networks that are traditionally difficult to measure, such as wait times, clinician churn, and time to follow-up after first visits. Additionally, CMS is seeking public input on creating the first national directory of health care professionals and services. A national directory would provide an opportunity to centralize data collection, auditing, and monitoring of network adequacy, and offer new insights into provider capacity across dynamic health plans and markets. Moreover, it would simplify current administrative burdens around this task, not only for health plans but also for clinicians, who must respond to numerous requests, timelines, and processes to update their information. Such standardization has the potential to improve the quality and accuracy of data currently featured in plan directories. A national directory may also facilitate new information collection, as when a patient reports network inadequacies in real time, as well as corrective action.

Finally, it is important for regulators to ensure that clinicians are incentivized to provide accurate and updated information, and that health plans are similarly incentivized to report and confront true inadequacies in their networks. Indeed, in cases where health professional shortages prevent plans from meeting network adequacy standards, corrective remediation, including through extension of telehealth services and guaranteed out-of-network coverage, should be applied. Strictly punitive actions alone could incentivize new plans to avoid serving patient populations or geographies that have historically raised network adequacy concerns.

The CMS’s new proposed Medicaid managed care rules mark an important shift toward strengthening network adequacy standards to address continued access gaps. Network adequacy regulation is not a panacea; clearly needed are broader efforts toward workforce expansion and improved network participation among clinicians with historically low rates of insurance acceptance, as in mental health care. But low-hanging interventions, including patient-focused metrics, accountability standards, and more reliable data inputs, will help ensure that network adequacy has meaning in the interim.
